# Zero-profile Hyperlordotic Spacer for Cervical Deformity Correction: Case Presentation and Technical Note

**DOI:** 10.7759/cureus.4097

**Published:** 2019-02-19

**Authors:** Paul R Krafft, Yusef I Mosley, Puya Alikhani

**Affiliations:** 1 Neurological Surgery, University of South Florida, Tampa, USA; 2 Neurological Surgery, Saint Luke's Hospital, Kansas City, USA

**Keywords:** zero-profile, hyperlordotic spacer, interbody, cervical deformity, kyphosis

## Abstract

Cervical spine deformity (CSD) can negatively affect the health-related quality of life (HRQOL) of patients, particularly the elderly, thus representing a socioeconomic problem of increasing importance. While surgical deformity correction has been linked to improved HRQOL, no universally accepted consensus exists for the operative management of CSD.

The authors demonstrate the feasibility of CSD correction, implementing anterior and posterior cervical osteotomies combined with the placement of multiple consecutive zero-profile hyperlordotic interbody spacers in a 55-year-old male with cervical kyphosis. This technique resulted in the satisfactory restoration of the patient’s cervical alignment and significantly ameliorated his presenting symptoms. The patient demonstrated maintained cervical lordosis and he remained symptom-free at the one-year follow-up.

The use of multiple consecutive zero-profile cervical interbody spacers can effectively and safely be utilized for the treatment of CSD. Further studies are needed to compare this technique with other standard surgeries used for CSD correction.

## Introduction

Cervical spine deformity (CSD) has become a serious medical and socioeconomic problem, predominantly affecting adult and elderly patients with underlying inflammatory disease, thoracolumbar malalignment, and those who have undergone previous cervical spine surgery, particularly multilevel laminectomies without posterior instrumented fusion [1-2]. Apart from potentially causing debilitating neck and back pain, CSD may be associated with impaired head mobility, dysphagia, and myelopathy, thereby affecting the overall functioning and health-related quality of life (HRQOL) of patients [3-4]. Despite its substantial impact on HRQOL, there is no universally accepted definition, grading system, or treatment strategy for this condition [5]. Indeed, CSD is broadly defined as an aberration of the physiologic lordotic alignment of the cervical spine; yet, discrepancies in the degree of cervical lordosis have been reported in healthy volunteers, depending on age, race, and method of evaluation [6-8]. Implementing a modified Delphi method, a panel of experts proposed a classification system for CSD [9]. The latter includes a deformity descriptor, such as “cervical,” “cervicothoracic,” “thoracic,” “coronal,” and “craniovertebral junction,” based on the apex of the deformity and five modifiers that incorporate sagittal, regional, and global spinopelvic alignment. While this classification system has demonstrated a satisfactory inter- and intraobserver reliability, correlations with patient outcomes are needed before it may be deemed clinically useful and applicable. While the operative correction of severe CSD may improve patients’ HRQOL, Smith et al. have demonstrated marked variations on preferred surgical approaches, level of osteotomies, and cervicothoracic instrumentation among experienced deformity surgeons [10]. This lack of consensus underlines the importance of further experiments evaluating patient outcomes following specific surgical approaches and treatment strategies. Surgical approaches for the correction of CSD include anterior-only, posterior-only, anterior followed by posterior, as well as posterior followed by anterior and posterior procedures. Common surgical techniques include anterior cervical discectomy and fusion (ACDF), anterior cervical corpectomy, anterior cervical osteotomy, Smith-Petersen and pedicle subtraction osteotomies, or any combination of the aforementioned [11]. Substantial complication rates have been documented following surgical CSD correction; however, due to advancement in anesthesia techniques, neuromonitoring, and spinal instrumentation, such operations have become more feasible. Smith et al. conducted a prospective multicenter study evaluating early complications for patients who underwent correction of CSD [12]. The overall complication rate was 43.6% and postoperative dysphagia was found to be the most common symptom following surgical CSD correction (11.5%).

The anterior cervical zero-profile vertebral interbody spacer is frequently used for the treatment of cervical spondylosis. Upon adequate placement, the implant is contained within the excised disc space and does not protrude past the anterior border of the adjacent vertebral body. Cortical screws secure it in place, thereby avoiding anterior plating. A recent meta-analysis demonstrated that zero-profile implants were associated with a significantly decreased incidence of postoperative dysphagia when compared to those that require anterior plating [13].

In 2017, NuVasive, Inc. (California, US) received U.S. Food and Drug Administration (FDA) 510 (k) clearance for the zero-profile CoRoent® Small Interbody^TM ^System to be used in cervical spine fusion involving up to four consecutive levels. Those interbody cages are manufactured from polyetheretherketone (PEEK) polymers offering a bone-like modulus, thus minimizing stress-shielding and promoting bone-healing. To our best knowledge, no reports have demonstrated the use of cervical zero-profile implants for CSD correction. We, therefore, present an innovative application of the zero-profile hyperlordotic device for CSD correction in a patient with severe cervical kyphosis.

## Technical report

Case presentation

**Figure 1:**
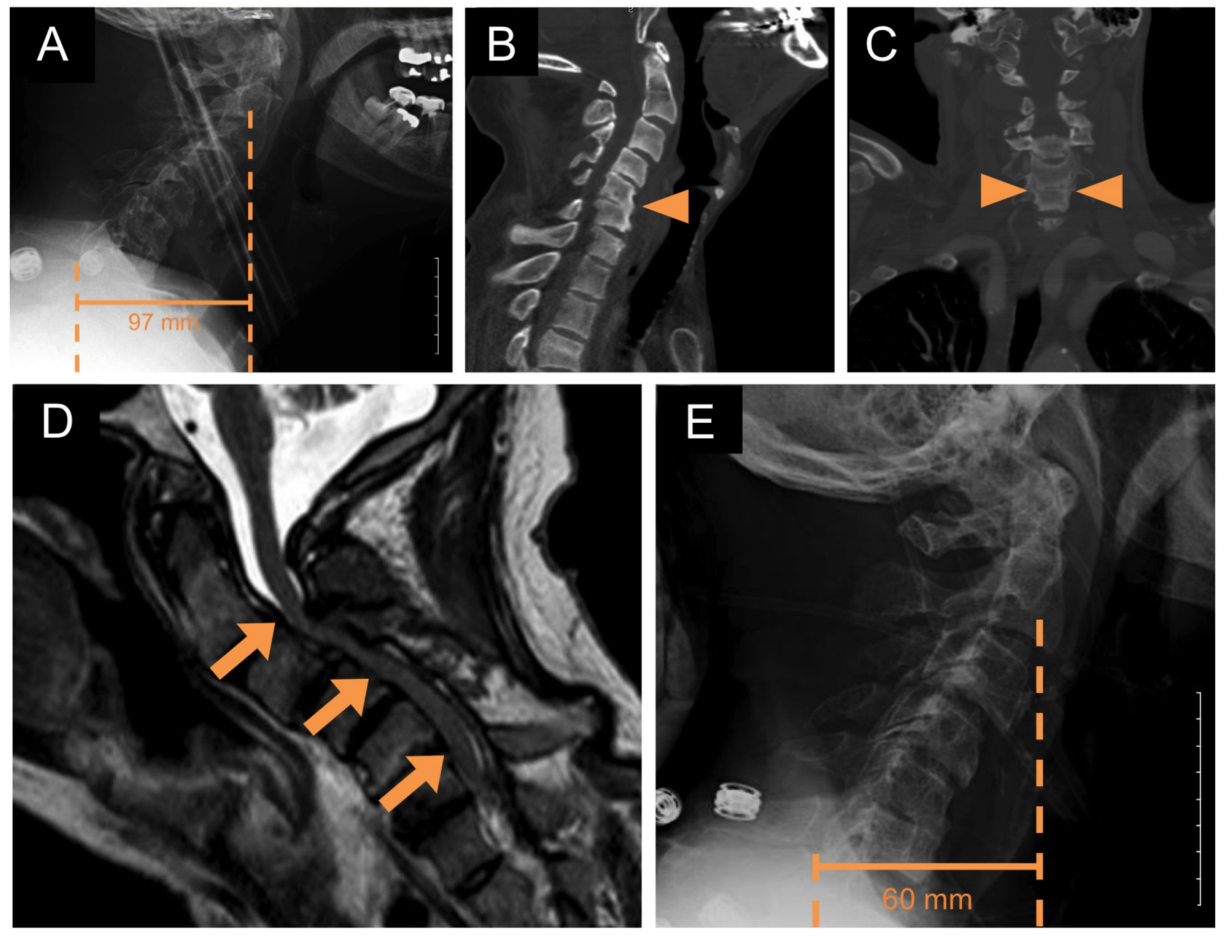
Preoperative imaging (A) Preoperative sagittal radiograph of the cervical spine demonstrating a kyphotic deformity with C2-C7 sagittal vertical axis (SVA) of 97 mm, Cobb angle of 1.3°, as well as multilevel degenerative disc disease with disc space narrowing (Scale bar: 50 mm). (B) Preoperative sagittal computed tomography (CT) of the cervical spine demonstrating autofusion at C5/6 (arrowhead). (C) Preoperative coronal CT angiogram of the neck demonstrating autofusion at C5/6 (arrowhead). (D) Preoperative sagittal non-contrast magnetic resonance imaging (MRI) of the cervical spine demonstrating significant central canal stenosis between C2/3 and C6/7 (arrows). (E) Sagittal radiograph of the cervical spine in traction (25 lbs) demonstrating correction of the kyphotic deformity with C2-C7 SVA of 60 mm, and Cobb angle of -13.4° of lordosis (Scale bar: 50 mm).

The patient is a 55-year-old male who presented to the emergency department after a ground-level fall. He complained of difficulty walking, impaired fine motor movements in both hands, severe neck pain, and inability to “look up.” His past medical history included long-standing hypertension, end-stage renal disease status post kidney transplantation, which was complicated by transplant rejection. He also reported a history of remote lumbar laminectomies for neurogenic claudication. On physical examination, the patient was alert and fully oriented. He was malnourished and in moderate distress, which was mostly attributable to his neck pain. His head was normocephalic and atraumatic. Cranial nerves 2-12 were grossly intact. He demonstrated 4+/5 strength in all major muscle groups of the right upper extremity. His left upper extremity demonstrated 4+/5 strength in all major muscle groups, except for deltoid function, which was graded 2/5. The strength is his bilateral lower extremities was graded 4/5. He demonstrated bilateral Hoffman’s signs and a bilateral four-beat clonus, as well as upgoing toes on plantar reflex testing. The following imaging studies were obtained: radiographs of the cervical spine, upright full-length scoliosis radiographs (not shown), as well as cervical, thoracic, and lumbar computed tomography (CT) scans, cervical CT angiogram, and magnetic resonance imaging (MRI). The lateral radiograph of the cervical spine demonstrated severe kyphotic deformity with a C2-C7 sagittal vertical axis (SVA) of 97 mm and a Cobb angle between C2 and C7 of 1.3 ° (Figure 1A). Furthermore, 4 mm of anterolisthesis of the C3 vertebral body with respect to C4 was noted as well as disc space narrowing at C3-C4, C4-C5, and C5-C6. A sagittal CT/CT angiogram of the cervical spine demonstrated anterior autofusion between C5/6 (Figure 1B and Figure 1C). Sagittal MRI of the cervical spine showed severe central canal stenosis between C2/3 and C6/7 (Figure 1D).

The patient was admitted to the neuroscience intensive care unit (ICU) where he was placed in 25 pounds of cervical traction using Gardner-Wells tongs. Cervical alignment improved. Specifically, lateral radiograph in traction demonstrated a C2-C7 SVA of 60 mm (Figure 1E) and a Cobb angle of -13.4° of lordosis. The patient was then taken to the operating room for multilevel ACDF, anterior osteotomies, followed by posterior osteotomies, and cervicothoracic fusion.

Technical note

**Figure 2:**
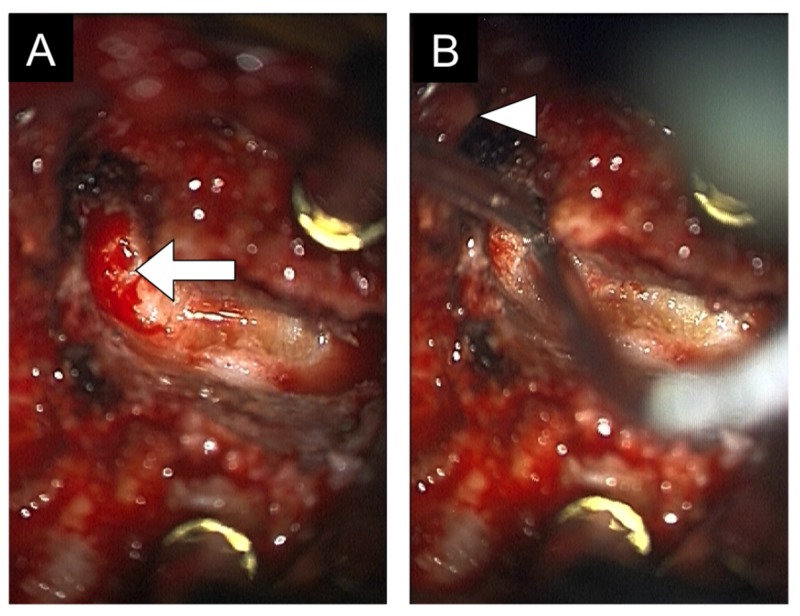
Intraoperative view (A) Intraoperative view through the microscope demonstrating the previously fused uncovertebral joint at C5/6, which was drilled opened (arrow). (B) Intraoperative view through the microscope demonstrating the opening of the transverse foramen and the exposure of the vertebral artery (arrowhead).

The patient remained supine and in cervical traction during anesthesia induction. Awake fiber-optic-aided endotracheal intubation was performed, and no neurofunctional changes were observed. General anesthesia was induced using intravenously administered titrated doses of propofol, ketamine, and remifentanil. Neuromonitoring electrodes (somatosensory-evoked potentials, motor evoked potentials, and electromyography) were placed in all appropriate muscle groups. Following that, fluoroscopy was used to localize the region of interest and a longitudinal skin incision was marked along the medial border of the sternocleidomastoid muscle (SCM), extending from C3 to C7. The surgical site was prepped and draped in the usual sterile fashion. The incision was made using a #10 blade. The platysma muscle was split and undermined. Soft connective tissue was bluntly dissected along the medial border of the SCM. The lateral border of the omohyoid muscle was then identified. A hand-held Cloward blade retractor (Millennium Surgical, Narberth, PA, US) was placed between the SCM and omohyoid muscle. The latter was retracted medically. Further blunt dissection exposed the bilateral Longus colli muscles, which were separated from the underlying vertebral body and disc space. A spinal needle was placed anteriorly into the exposed disc space and lateral fluoroscopy was used to confirm the correct level. The dissection of the Longus colli muscles was carried out between the inferior endplate of C3 and the superior endplate of C7. Following that, the endotracheal tube cuff was deflated for the remainder of the procedure. Next, Caspar pins were placed centrally in the C3 and C4 vertebral bodies and the suitable cervical distractor (Aesculap Implant Systems, PA, US) was used to distract the C3/4 disc space. A standard discectomy was performed at that level. Briefly, the annulus was incised using a bayoneted annulotomy knife and the disc material was removed by the use of pituitary rongeurs, curettes, and rasps. Following that, a Grade 1 anterior osteotomy was performed as previously described [14]. We used a Midas Rex MR7 high-speed pneumatic drill (Medtronic PLC, Minnesota, US) to complete a partial uncovertebral joint resection, followed by a partial facet joint resection. The zero-profile hyperlordotic cage was placed into the disc space under fluoroscopic guidance. Two 3.5 mm x 14 mm screws were placed through the inferior endplate of C3. The cervical distractor system was then moved to the C6/7 level and the above procedure was repeated. At this level, the intervertebral spacer was secured with 2 3.5 mm x 14 mm screws that were placed through the superior endplate of C7. Next, attention was directed to the C4/5 and C5/6 levels, which were found to be autofused. Grade 4 osteotomies were performed, which include an anterior bony resection through the lateral vertebral body and the uncovertebral joints into the transverse foramen (Figure 2) [14]. For the resection of the uncovertebral joints, we utilized a rough-cutting diamond drill bit with a copious amount of irrigation to minimize the risk of vertebral artery injury. Similar to the other cervical levels, zero-profile hyperlordotic interbody cages were placed at C4/5 and C5/6. Hemostasis was achieved using bipolar cautery. A Hemovac drain was placed in the surgical bed and the wound was sutured in multiple layers.

The patient was then rotated 180° into a prone position and the cervical and upper thoracic spine was exposed using subperiosteal dissection with monopolar cautery. The starting points for the C2 pars screws, the C3 to C6 lateral mass screws, and the T1 and T2 thoracic pedicle screws were identified, marked, and prepared for hardware placement. Grade 2 osteotomies (Ponte osteotomies) were performed at C6-C7, which involved the resection of the spinous process, lamina, facet joint, and associated ligaments at the aforementioned levels [14]. Following that, 3.5 mm x 18 mm screws were placed in the bilateral pars of C2, 3.5 mm x 14 mm screws were placed in the bilateral lateral masses of C3 through C6, and 4.5 mm x 30 mm screws were placed into the bilateral pedicle of T1 and T2. Bilateral 4.0 cobalt chrome rods were used to connect the screws. A cross-connector was inserted to increase the stability of the construct (all spinal hardware was supplied by NuVasive). A Hemovac drain was placed in the surgical bed and the wound was closed in multiple layers.

**Figure 3:**
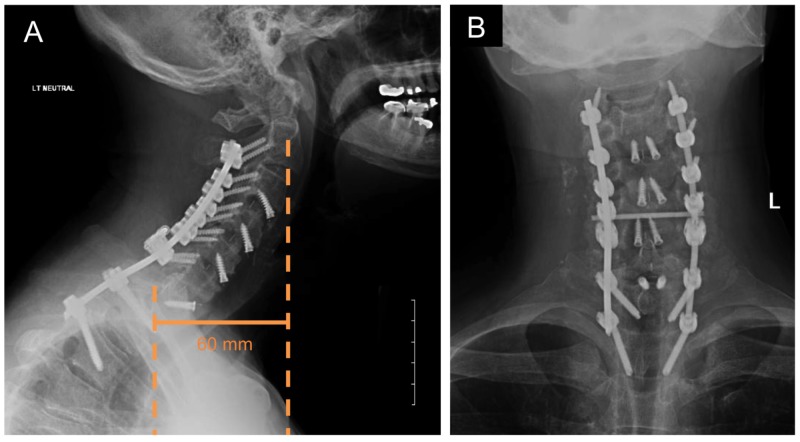
Postoperative imaging (A) Postoperative sagittal radiograph of the cervical spine demonstrating satisfactory cervical alignment with C2-C7 sagittal vertical axis (SVA) of 60 mm, Cobb angle of - 31.7° of lordosis. Cervicothoracic hardware remained in place and intact (scale bar: 50 mm). (B) Postoperative coronal radiograph of the cervical spine demonstrating intact hardware.

The patient tolerated the procedure well, his myelopathy resolved completely, and he was able to ambulate independently on postoperative Day 2. A postoperative lateral radiograph demonstrated good cervical alignment (C2-C7 SVA: 60 mm, Cobb angle: -31.7° of lordosis), and the patient continued to do well in follow-up at one year after surgery (Figure 3).

## Discussion

The main objectives of CSD correction surgery include: (1) decompression of neuronal and vascular structures in the neck, (2) restoration of cervical alignment permitting sufficient horizontal gaze, (3) spinal stabilization, and (4) meticulous arthrodesis in order to prevent the formation of pseudoarthrosis while minimizing surgical complications such as dysphagia and neurofunctional deficits [11]. Multiple surgical techniques for CSD correction exist, including anterior, posterior, or combined strategies. Since there is no universally accepted consensus regarding which operative method to use for any given case, selecting the best approach that ensures the optimal clinical outcome can be challenging [10]. Severe kyphotic deformity of the cervical spine can be successfully addressed with combined approaches that include anterior discectomies and osteotomies with posterior osteotomies and cervicothoracic instrumentation. Anterior cervical plates are frequently utilized to keep the intervertebral spacer in place; however, extensive anterior plating is associated with higher incidences of postoperative dysphagia and, furthermore, limits the ability to achieve posterior alignment correction [13]. Anterior plating is obsolete when using a zero-profile intervertebral spacer since these devices anchor into the superior and/or inferior endplates of the adjacent vertebral bodies via cortical screws. This potentially may not only decrease postoperative dysphagia but also make these devices suitable for combined approaches for deformity correction. While zero-profile devices are frequently utilized for the management of cervical spondylosis, their application may be extended to include cervical kyphosis correction, especially since receiving FDA approval for the use in up to four consecutive levels. These intervertebral spacers are available in a hyperlordotic form, which aids in CSD correction as well.

## Conclusions

In summary, we have described the successful application of a multilevel, hyperlordotic, zero-profile intervertebral spacer for CSD correction. We believe that these techniques can be utilized safely and effectively in combined approaches to the cervical spine. Further clinical studies are needed to establish this method as a standard for CSD correction surgery.
